# The Fetal Safety of Angiotensin Converting Enzyme Inhibitors and Angiotensin II Receptor Blockers 

**DOI:** 10.1155/2012/658310

**Published:** 2011-12-13

**Authors:** Myla E. Moretti, Daniela Caprara, Irina Drehuta, Emily Yeung, Stefanie Cheung, Lisa Federico, Gideon Koren

**Affiliations:** The Motherisk Program, The Hospital for Sick Children, 555 University Avenue, Toronto, ON, Canada M5G 1X8

## Abstract

Angiotensin converting enzyme (ACE) inhibitors and angiotensin II receptor blockers (ARBs) are known to cause fetal renal damage in pregnancy. Due to conflicting reports in the literature, their safety after first trimester exposure has been debated. Our aim was to determine whether the use of ACE inhibitors or ARBs in the first trimester of pregnancy is associated with an increased risk for major malformations or other adverse outcomes. All subjects were prospectively enrolled from among women contacting a teratogen information service. At initial contact, details of maternal medical history and exposures were collected and follow-up interviews were conducted to ascertain pregnancy outcomes. Two comparator groups, women with hypertension treated with other antihypertensives, and healthy controls were also recruited. Baseline maternal characteristics were not different among the three groups. There were no differences in rates of major malformations. Both the ACE-ARBs and disease-matched groups exhibited significantly lower birth weight and gestational ages than the healthy controls (*P* < 0.001 for both variables). There was a significantly higher rate of miscarriage noted in the ACE/ARB group (*P* < 0.001). These results suggest that ACE inhibitors/ARBs are not major human teratogens; however, they may be associated with an increased risk for miscarriage.

## 1. Background

Hypertension is a fairly common condition, estimated to affect between 6% and 8% of pregnancies [1]. It can occur as one of four conditions: chronic hypertension, preeclampsia-eclampsia, chronic hypertension with superimposed preeclampsia, and gestational hypertension [2]. Hypertension is associated with an increased risk of adverse effects in both the mother and the fetus, and treatment is warranted. Perinatal and infant complications may include prematurity, neonatal death, placental abruptions, and small-for-gestational age babies [3–7]. Maternal complications include pulmonary edema, hypertensive encephalopathy, retinopathy, cerebral hemorrhage, and acute renal failure [2], which are worse in untreated patients. 

Data on the safety of antihypertensive drugs in pregnancy are relatively sparse [8]. Based on the existing data, methyldopa, nifedipine, labetolol, and other beta-blockers have been considered the drugs of choice in the treatment of hypertensive disorders in pregnancy [9]. 

Angiotensin converting enzyme inhibitors (ACE) are now widely used as first-line medications in nonpregnant hypertensive patients. A more recent class of agents, the angiotensin II receptor blockers (ARBs) are also gaining in popularity. Unfortunately, both of these classes of drugs have been contraindicated in pregnancy because of their association with characteristic adverse fetal effects [9] when used beyond the first trimester of pregnancy, including fetal hypocalvaria and renal insufficiency. The etiology of these defects appears to be related to fetal hypotension and reduced renal blood flow in the fetus.

Intrauterine growth restriction, prematurity, patent ductus arteriosus, severe neonatal hypotension, neonatal anuria, and neonatal or fetal death have also been observed with these drugs [10]. Anuria associated with oligohydramnios may produce fetal limb contractures, craniofacial deformities, and pulmonary hypoplasia. Based on their similar pharmacologic effects, it is generally assumed that the ARBs will behave in much the same manner although published data on large numbers of exposed pregnancies do not exist [11–19]. To date, most human cohort studies or case series have failed to find teratogenic effects of ACE inhibitors after first trimester exposure [11, 20–24]. Recently, based on a relatively small cohort study, Cooper et al. suggested an increased risk of cardiovascular effects with first trimester use of these agents [25]. These findings, if real, are of major concern, because ACE and ARBs continue to be used in women of reproductive age, many of whom may use inadequate contraception [26, 27]. Moreover, since half of all pregnancies are unplanned [28, 29], inadvertent exposures to ACE and ARBs in pregnancy will continue to occur.

The primary objective of this study was to determine the risk for major congenital malformations following maternal exposure to ACE inhibitors and ARBs during the first trimester of pregnancy. 

## 2. Methods

This was a prospective, observational, controlled cohort study. Eligible women were identified among callers to the Motherisk Program at the Hospital for Sick Children in Toronto. The Motherisk Program is a counseling service for women, their families, and health professionals on the safety or risk of drugs, chemicals, radiation, and infection during pregnancy. The study groups consisted of women who contacted the Motherisk Program concerning exposure to ACE-ARBs during the first trimester, other antihypertensives in pregnancy and healthy comparators not exposed to any known teratogen or medications for chronic conditions. Any patient reporting use of ACE/ARBs into pregnancy was eligible for inclusion. We controlled for potential effects of hypertension by comparing this group to two other groups of subjects, women exposed to other antihypertensive agents (including methyldopa or calcium channel blockers), and a healthy group without hypertension. The comparator groups were matched to the study groups by gestational age at recruitment, maternal age, and alcohol and cigarette use. The healthy comparator group was also selected from among callers to the Motherisk program. These women had no chronic medical conditions. Subjects were excluded if they were unwilling or unable to complete the follow-up interviews in English.

At initial contact with the patient before or during the early weeks of pregnancy, standardized questionnaires were used to document maternal medical history and exposures. Information about current and past pregnancies was obtained as was details about concurrent medical conditions. After the expected date of delivery, patients were followed up; the information collected at intake was complimented with additional details on medical conditions or exposures occurring since the initial contact. Details about delivery and infant outcomes were also recorded at follow-up.

The primary outcome measure was the rates of major malformations, which was compared among the 3 groups. Secondary endpoints included live birth rates, birth weight, gestational age, rates of perinatal, and neonatal complications as well as rates of miscarriage. Possible confounding factors, such as the presence of diabetes, were also considered in the analysis. 

The rates of major malformations among the 3 groups were compared using the chi-square test or Fisher's exact test as appropriate. Continuous data were compared using the Kruskal-Wallis analysis of variance for three group comparisons. An alpha level of 0.05 was considered statistically significant and two-tailed tests were used for all analyses. Statistical analysis was performed with SigmaStat software (version 3.0). 

This study was approved by the Research Ethics Board of the Hospital for Sick Children. 

## 3. Results

We were able to successfully collect and follow 138 pregnancies exposed to ACE inhibitors or ARB's, 112 pregnancies exposed to “other” antihypertensives and 138 healthy pregnancies. There were no significant differences among the three groups in terms of maternal characteristics (Table 1), as the ACE/ARB group was matched with the healthy comparator group on most of these parameters. In the ACE/ARB group, the majority of women were exposed to these drugs exclusively in the first trimester (114 women—90%). A total of 8 (6.3%) women were exposed to these drugs in the first and second trimester, while only 6 (4.7%) continued the drugs for all three trimesters. The ACE/ARB group included 38 (27.5%) women exposed to ramipril, 25 (18.1%) exposed to lisinopril, and 15 (10.9%) women exposed to enalapril (Table 2). In the ACE/ARB group, there were 18 diabetics (13%), 6 with Type 1 diabetes mellitus and 12 with Type II diabetes mellitus.

When comparing pregnancy outcomes (Table 3), there were no differences in sex of offspring or rates of fetal distress. There were significant differences in birth weight and gestational age at delivery with both the hypertensive patient groups exhibiting lower birth weights (3225 g ACE/ARB group, 3063 g other antihypertensives, and 3511 g healthy controls, *P* < 0.001) and earlier gestational ages at delivery (37.6 weeks ACE/ARB group, 37.8 weeks other antihypertensives, and 39.6 weeks healthy controls, *P* < 0.001) compared to the healthy controls. There was a significantly higher rate of miscarriages in the ACE/ARB group, as compared to the “other” antihypertensive and healthy control groups (18.0%, 8.9%, and 11.8%, resp., *P* < 0.001).

There were 2 cases of major malformations in each of the three groups (Table 2), with no statistical differences among them (*P* = 0.99). As there was not a higher rate of malformations in the exposed group, we did not perform detailed analysis with diabetes as a covariate; however, one malformed case in the treatment group was from a diabetic mother. In addition, among the ACE/ARB group two of the spontaneous abortions occurred in diabetic mothers. Analyzing the pregnancy outcomes excluding these two cases did not change the significance in the rate of spontaneous abortions.

## 4. Discussion

Establishing the safety of ACE inhibitors and ARBs after first trimester exposure is important for a number of reasons. Most notably is that women continue to need effective treatment for their existing chronic hypertension and that a large number of pregnancies will be exposed inadvertently to these agents. Accurate information on the safety of these agents will assist women and their health care practitioners in making rational choices about appropriate treatment. While there is a consensus that ACE inhibitor/ARBs should be discontinued when pregnancy is diagnosed to prevent fetal renal damage and associated complications, women often do not plan pregnancy, and fetal exposure in the first trimester is inevitable.

Our results are reassuring and consistent with a growing body of evidence that did not find an apparent increased risk for malformations among liveborns following exposure to ACE inhibitor/ARBs in early pregnancy [11]. In fact, the rates of malformations were comparable to our healthy comparator group. Given that the ACE/ARBs are known to affect the fetal renin-angiotensin axis which becomes active in the second trimester, it is not surprising that to date, adverse fetal effects of these agents have been shown only after exposures which continued into the second half of pregnancy. Our findings support the current hypothesis that teratogenic effects are likely mediated through disruptions in the renin-angiotensin axis and, therefore, not observed with such early exposures.

There were significantly more spontaneous abortions in the ACE/ARB group as compared to the other antihypertensive or healthy groups. Some animal data support this finding showing an increase in mortality among fetuses exposed during organogenesis [30]. This may also be the result of confounding effects of underlying maternal conditions, including higher incidence of diabetes mellitus among women receiving ACE/ARBs [31], but the possibility of spontaneous abortions as a result of fetal malformation cannot be excluded. In addition, while the number of diabetics with spontaneous abortion was small, precluding our ability to perform a regression analysis, excluding these two cases for a subgroup analysis did not change the significant findings of the spontaneous abortion outcome. 

In the three-way comparison, there was a significant difference in mean gestational age at birth as well as birth weight, with babies born either to mothers exposed ACE/ARBs or to other antihypertensive drugs exhibiting a lower mean gestational ages at birth as well as a lower birth weights. The decrease in gestational age at birth as well as birth weight is consistent with the findings in women with chronic hypertension [32], attributable to placental dysfunction and decreased placental blood flow [30]. Including a disease-matched comparison group, our data suggest that the decrease in gestational age at birth and the lower birth weight are likely related to disease effects similar to all previous studies. Our ability to detect small increases in the risk of major malformations is limited by the available sample size. Though this cohort has an 80% power (with *α* = 0.05) to detect only a 2.5-fold increased risk, however, our data are in agreement with several recent published cohort studies and series [11, 20–24, 33], which failed to show increased malformation rates after first trimester exposure to ACE inhibitors/ARBs. In addition, we were unable to provide analysis on any particular ACE/ARB, to determine if there are differential effects of the two classes of drugs, or to assess for dose effects as the numbers in any particular drug-dose combination were small. 

Our study may be limited by population selection bias. Namely, subjects were recruited following contact with a teratogen information service and may not represent the general antihypertensive using population. These are patients who have sought out additional information about their risks may be more diligent about seeking out prenatal or medical care in general. 

The positive study by Cooper et al. [25] has been heavily criticized for inappropriately addressing potential confounders such as diabetes [31], some of which may not have been diagnosed. A large study by Malm et al. suggest that the apparent increased risk of ACE inhibitor is the result of maternal diabetes, as exposure to ACE inhibitors without diabetes was not associated with a higher teratogenic risk [33]. While we had insufficient cases to rule out a possible confounding effect of diabetes in our cohort, we had a substantial proportion of subjects with underlying diabetes, and it is apparent that subjects on ACE/ARBs are more likely to be diabetics than those on other antihypertensives. 

Our findings suggest that inadvertent exposure to ACE inhibitors/ARBs in the first trimester of pregnancy may not present significant risks for malformations in live births but may be associated with higher rates of spontaneous abortion. However, given the strong evidence for teratogenicity beyond the first trimester and the availability of other safer effective antihypertensives in pregnancy, it is imperative that women on such agents receive prompt attention in the early part of pregnancy so that their antihypertensive medications can be appropriately adjusted.

## Figures and Tables

**Table 1:** Characteristics of included subjects.

Characteristic	ACE/ARB exposed (*n* = 138)	Other antihypertensives (*n* = 110)	Healthy nonexposed (*n* = 138)	*P* value
Age (yrs ± SD)*	34.9 ± 4.9	34.3 ± 4.2	33.9 ± 4.5	0.18
Gravidity (%)^†^				
1 2 ≥3	41 (29.7) 39 (28.3) 58 (42.0)	31 (28.2) 41 (37.3) 38 (34.5)	41 (29.7) 41 (29.7) 56 (40.6)	0.60
Parity (%)^†^				
0 1 ≥2	59 (42.7) 38 (27.5) 41 (29.7)	44 (40.0) 46 (41.8) 20 (18.2)	57 (41.3) 47 (34.1) 34 (24.6)	0.13
Previous miscarriage (%)^†^				
0 1 ≥2	110 (79.7) 17 (12.3) 11 (8.0)	81 (73.6) 22 (20.0) 7 (6.4)	105 (76.1) 21 (15.2) 12 (8.7)	0.54
Previous elective abortions (%)^†^				
0 ≥1	119 (86.2) 19 (13.8)	103 (93.6) 7 (6.4)	123 (89.1) 15 (10.9)	0.27
Gestational age at call (wks ± SD)^‡^	7.0 ± 3.4	10.5 ± 8.3	7.4 ± 3.4	0.09
Alcohol^†^				
No Light	114 (85.1) 20 (14.9)	100 (90.1) 11 (9.9)	125 (90.6) 13 (9.4)	0.30
Smoking^†^				
No Yes	117 (88.0) 16 (12.0)	102 (93.6) 7 (6.4)	129 (93.5) 9 (6.5)	0.18

*One-way Anova, ^†^chi-square test, ^‡^one-way Anova on ranks.

**Table 2:** Specific ACE/ARB's used by expose subjects.

	Count (%)
Ramipril	38 (27.5%)
Lisinopril	25 (18.1%)
Enalapril	15 (10.9%)
Monopril	8 (5.8%)
Valsartan	8 (5.8%)
Perindopril	7 (5.1%)
Candesartan	6 (4.3%)
Irbesartan	6 (4.3%)
Losartan	5 (3.6%)
Quinapril	5 (3.6%)
Cilazapril	3 (2.2%)
Fosinopril	3 (2.2%)
Telmisartan	3 (2.2%)
Captopril	2 (1.4%)
Prinivil	1 (0.7%)
Trandolapril	1 (0.7%)
Polytherapy	2 (1.4%)

**Table 3:** Pregnancy outcomes following exposure to ACE/ARBs or other antihypertensives as compared to a healthy comparator group.

Characteristic	ACE/ARB exposed (*n* = 139^a^)	Other antihypertensives (*n* = 112^b^)	Healthy nonexposed (*n* = 138)	*P* value
Fetal outcome				
Livebirth Spontaneous abortion Elective abortion Fetal death	108 (77.7%) 25 (18.0%) 6 (4.3%) 0	105 (93.7%) 4 (8.9%) 0 3 (2.7%)	120 (88.2%) 16 (11.8%) 0 0	<0.001
Gestational age at birth (wks ± SD)	37.6 ± 3.1	37.8 ± 2.8	39.6 ± 1.6	<0.001
Delivery				
Vaginal Cesarean section	62/108 (57.4%) 46/108 (42.6%)	57/106 (53.8%) 49/106 (46.2%)	83/120 (69.1%) 37/120 (30.8%)	0.045
Preterm delivery				
No Yes	81/108 (75%) 27/108 (25%)	79/105 (75.2%) 26/105 (24.8%)	117/120 (97.5%) 3/120 (2.5%)	<0.001
Birth weight (grams ± SD)	3225 ± 862	3063 ± 839	3511 ± 471	<0.001
Sex				
Male	59 (54.6%)	49 (46.6%)	57 (47.5%)	0.43
Fetal malformations^†^				
Yes	2/108 (1.8%)	2/105 (1.9%)	2/120 (1.6%)	0.99

^a^Including 1 twin pregnancy.

^b^Including 2 twin pregnancies.

^†^Fetal malformations are reported as a proportion of liveborn (in the ACE/ARB group-1 choanal atresia and 1 hypospadias, in the other antihypertensives group-1 unspecified heart murmur and 1 undescended testicle, in the health unexposed group-1 Down's syndrome and 1 inguinal hernia).
